# Leveraging a Joint learning Model to Extract Mixture Symptom Mentions from Traditional Chinese Medicine Clinical Notes

**DOI:** 10.1155/2022/2146236

**Published:** 2022-03-08

**Authors:** Yuxin Sun, Zhenying Zhao, Zhongyi Wang, Haiyang He, Feng Guo, Yuchen Luo, Qing Gao, Ningjing Wei, Jialin Liu, Guo-Zheng Li, Ziqing Liu

**Affiliations:** ^1^The Third Affiliated Hospital, Henan University of Chinese Medicine, Zhengzhou 450046, China; ^2^Information Office, Henan University of Chinese Medicine, Zhengzhou 450046, China; ^3^School of Computer Science, South China Normal University, Guangzhou 510631, China

## Abstract

This paper addresses the mixture symptom mention problem which appears in the structuring of Traditional Chinese Medicine (TCM). We accomplished this by disassembling mixture symptom mentions with entity relation extraction. Over 2,200 clinical notes were annotated to construct the training set. Then, an end-to-end joint learning model was established to extract the entity relations. A joint model leveraging a multihead mechanism was proposed to deal with the problem of relation overlapping. A pretrained transformer encoder was adopted to capture context information. Compared with the entity extraction pipeline, the constructed joint learning model was superior in recall, precision, and F1 measures, at 0.822, 0.825, and 0.818, respectively, 14% higher than the baseline model. The joint learning model could automatically extract features without any extra natural language processing tools. This is efficient in the disassembling of mixture symptom mentions. Furthermore, this superior performance at identifying overlapping relations could benefit the reassembling of separated symptom entities downstream.

## 1. Introduction

Clinical notes are collected by physicians during the process of clinical services, as part of the Patient Experience Data (PED) [1]. As a significant component of “real-world evidence,” PED plays a pivotal role in the efficacy evaluation towards complementary and alternative medicine like Traditional Chinese Medicine (TCM). Rather than general laboratory experiments, TCM is based on clinical practice and clinical experiments [2]. Due to TCM's personalized nature, the efficacy of most TCM therapies and prescriptions cannot be verified without practical accounts. Meanwhile, as a patient experience-oriented medical science, disease progression and reversion are recorded concisely. This offers essential references for efficacy evaluation. To make full use of TCM therapy and mitigate risk, many studies have emphasized distilling practical and repeatable clinical strategies from clinical cases that have shown to be effective on common diseases like cancer [3, 4]. To support this research, a corpus of free-text clinical notes needs to be transformed into structured data. This is both labor-intensive and time-consuming [5]. Hence, an increasing number of studies have addressed the problems of medical information extraction.

Distinct from Western medicine, TCM clinical notes focus on symptoms, syndromes, formulae, and herbs [6]. As the core component of patient feedback, symptoms, and their changes, tend to relate directly to the efficacy evaluation. However, symptom descriptions in TCM clinical notes complicate entity extraction. Ancient Chinese was dominated by single-character words, while most modern Chinese words consist of two or more characters. This phenomenon has evolved over a long time horizon, and even at present, the border between phrases and compound words in Chinese remains blurry. This leads to the problem of word separation [7].

Ancient TCM literature retains the expression of symptoms and their descriptions. In notes in modern TCM records, the symptom often appears in the form of compound words/phrases with subordinate or coordinate structures containing multiple symptoms and their severity, which are regarded as separable words. In the natural language processing (NLP) context, these are called “nested entity recognition”. However, most of the existing frame of nested entity recognition is focused on the recognition of continued entity mentions while Chinese clinical context abounds with noncontinuous named entities. For instance, “头身强痛” (head, trunk, heavily, hurt) should be disassembled into “头强痛”and “身强痛”. A more common expression of the two symptoms would be “头痛” (headache) or “身痛” (trunk pain). “强” (heavily) as an adverb of degree describes the symptom and also needs also be considered because changes could happen in the following treatment. The symptom degree variance could then be an indicator of the efficacy. To distinguish our work from the conventional NLP shared task, we denote the problem as “mixture symptom mentions,” in which the noncontinuous entity mention recognition would account for a large proportion.

**Figure 1:**
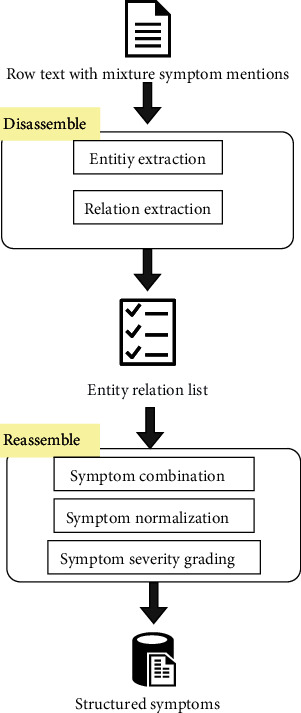
The framework for extracting mixture symptom mentions.

To extract symptoms expressed in full from TCM clinical notes necessitates a two-step method. First, the mixture symptom mentions need to be disassembled. Second, the separate parts need to be reassembled into complete symptom mentions. This paper focuses on the first part, leveraging the entity relations extraction to disassemble the mixture symptom mentions. As shown in Figure 1, three entity types and two relation types are defined as the extraction targets. An end-to-end model was leveraged to accomplish this task. This paper's contributions can be summarized as follows:

1. A dissemble-reassemble method is proposed to solve the mixture problem of TCM symptom extraction
2. A TCM-adapted transformer encoder is fine-tuned based on an existing pretrained model
3. An end-to-end entity relation joint learning model is constructed to prevent the error delivery that could happen in a conventional extracting pipeline
4. A multihead selection mechanism processes the relation overlapping

## 2. Related work

Driven by increasing need for structured clinical data, significant progress has been made on extracting entity and relation from unstructured clinical text. There are two approaches to extracting entity and relation—extraction pipeline and joint learning methods. The entity relation extraction pipelines divide the task into two parts. They first conduct entity recognition and then classify the relations between each entity pair. The joint learning methods, which utilize an end-to-end model to combine two tasks through specific strategies, extract entity and relations in one pass.

Due to the variety of traits, the extraction pipeline structures are just as diverse. Many researchers have chosen the pipeline approach [8–12] to detect entities and then extract relations between them. For instance, Vashishth et al. [10] and Hoffmann et al. [12] employed external components or knowledge-based methods to assist the relation extraction. Considerably more researchers have utilized deep neural networks to achieve superior performance [8, 9, 11]. This has led to research into the modification and optimization of deep neural network-based models. However, the extraction pipelines may cause error propagation problems or ignore the correlations between entity recognition and relation extraction. This would diminish extraction performance.

Joint learning models have been proposed to address the disadvantages of extraction pipelines. Miwa and Bansal [13] constructed bidirectional tree-structured RNNs to obtain dependency information from sentences. This was the first time that a neural network has been used for joint extraction of entity and relation. Zheng et al. [14] have proposed a novel tagging scheme to extract entity and relation, which is to convert the joint extraction task to a tagging problem. To solve the problem of the overlapping relation, Bekoulis et al. [15] regard the joint extraction of entity and relations as a multihead selection problem. Takanobu et al. [16] propose a joint extraction model based on reinforcement learning. While applying joint learning structures to relation extraction is becoming popular, part of the concern over relation extraction is transformation to the relation overlapping processing. This paper is one of these studies that assess the joint learning model on the overlapping relation extraction, in the context of clinical narrative.

## 3. Materials and Methods

### 3.1. Materials and Annotation

**Figure 2:**
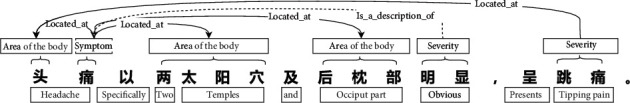
An example of annotated sentences.

According to observations, TCM mixture symptom mentions tend to involve three medical entities, 部位 (area of the body), 症状 (symptom), and 程度 (severity). The relation between them can be attributed to two classes, “位于 (located_at)” and “描述 (is_a_description_of).” As shown in Figure 2, the entity and relations above are set as the extraction target.

In this study, we collected 10,000 clinical notes from Professor Zhang Lei, a renowned TCM master in China. These clinical notes recorded nearly ten years of Professor Zhang's clinical practice, detailing chief complaints, medical history for the present illness, anamnesis, personal history, family history, diagnosis, TCM differentiation, and prescription. Over 1,000 patients were involved. For the training of the proposed entity relation extraction model, 2,255 clinical notes from 2006 comprised the annotated corpus. ATCM M.M. was engaged full-time in the corpus annotation and revision.

Table 1(a) shows that 72,894 entities were annotated in the collected corpus. A majority of the entities were of type “症状” (symptom), one-third were “部位” (area of the body), and 7% were “程度” (severity).  Table 1(b) reveals that 41,246 entity relations were annotated in total, 35,804 of which were of type “位于” (located_in), 87% of all relations; the remaining 5,442 relations were of type “描述” (is_a_description_of).

In accordance with the defined schema, all entities pertaining to “部位” (area of the body) and “程度” (severity) were involved in one or several relations, while “症状” (symptom) could either come along with two other entity types or appear individually. As displayed in Tables 2 and 3, 706 of the 41,004 named entities were not involved with any relations. This was only 11.5% of the population. 16% of the entities appeared in at least two relations, which means that relation overlap was common within the constructed corpus.

## 4. Methods

### 4.1. Pretrained Character-Level Embedding for TCM

The bidirectional encoder representations from transformers (BERT) [17] is a pretrained representation model, noted for its strength in contextual word representation. Utilizing the masked language model, we fine-tuned the pretrained “BERT-base-Chinese” model to the corpus of nearly 50,000 free-text TCM clinical notes. The acquired character-level representation would be fed into downstream extraction tasks.

### 4.2. Relation Extraction Pipeline

In the relation extraction pipeline, the entity named recognized model and the relation extraction model were built separately. In the present study, we first applied the outperformed bidirectional long-short time memory and conditional random fields (bi-LSTM-CRF) framework [18, 19] to extract the symptom entities. Then, along with the obtained entities, the sentence texts would be sent into a bidirectional gated recurrent unit (bi-GRU) [20, 21] to extract the relations.

### 4.3. Joint Learning Model

To solve the problem of relation overlapping, we build a joint model that is not only able to simultaneously extract the entities and the possible relations, but which also relies on neither manual features nor NLP tools [15]. The model was built in the frame of the multihead selection problem which assumes that any particular entity may be involved in multiple relations with other entities. First, the sentence sequence was input. Then, the entity label, relation type, and head (the last token of factual subject entity) of the corresponding relations were output.

**Figure 3:**
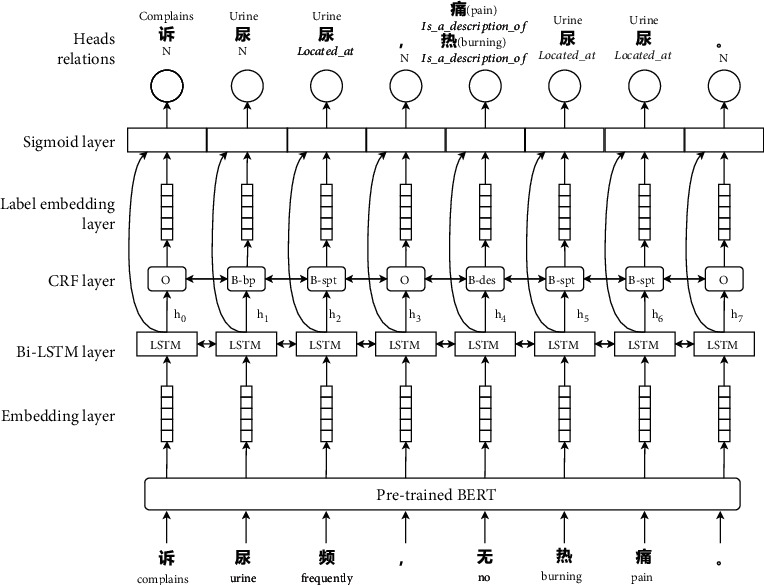
The joint model framework for entity relation extraction.

As shown in Figure 3, the model was constructed with five components. The first component is an encoding layer, transforming the input sequence to character-level embedding. The second and third components constitute the bi-LSTM layer, followed by a linear chain-controlled random field (CRF) layer, in charge of the name identity recognition. The fourth part is the label embedding, which takes the entity tags as input, and outputs learned label embedding representation that encodes the information from the named entities. It then applies them to relation extraction. The last part is the sigmoid layer for the multihead probable scoring.

Given a sentence *S*_char_ = [char_1_, char_2_, ⋯, char_*n*_] as a sequence of tokens, a pretrained BERT was utilized to map each token to a character vector. The bi-LSTM and CRF layers as a conventional NER module receive the character vectors as input and then output the predicted entity label and the bi-LSTM hidden states $h_{i}=\left[{\overset{⟶}{h}}_{i},\;{\overset{⟵}{h}}_{\text{I}}\right]$. The NER module was trained by minimizing the cross-entropy loss *L*_ner_.Through the label embedding layer, the predicted entity label char_*i*_ is represented as vector *g*_*i*_ and concatenated with the bi-LSTM hidden state. The output of token char_*i*_ in the NER phase *z*_*i*_ = [*h*_*i*_, *g*_*i*_] becomes the input of the relation extraction module.

The relation extract module was formulated as a multihead selection problem. Head denotes the last token of an entity; multihead means the assumption that every entity might have a relationship with any other entities. Given that the relation label set *R* and the token vectors char_*i*_ and char_*j*_ belong to *S*_char_, the multihead selecting model predicts that the probability of these two is in the relation of labels that belong to *R*. Given a relation label *r*_*k*_, the relation score can be calculated as

(1) $$\begin{array}{c} s^{\left(r\right)}\left(z_{j},z_{i},r_{k}\right)=V^{\left(r\right)}f\left(U^{\left(r\right)}z_{j}+W^{\left(r\right)}z_{i}+b^{\left(r\right)}\right). \end{array}$$

As mentioned above, *z*_*j*_ and *z*_*i*_ are the NER phase outputs of token char_*i*_ and char_*j*_, respectively. Through dimension reduction and activation, the obtained score was transformed to the probability of token char_*j*_ to be selected as the head of  char_*i*_ and with the relation label *r*_*k*_:

(2) $$\begin{array}{c} \mathrm{Pr}\left(\text{head}=\text{cha}{\text{r}}_{j},\text{label}=r_{k}\;|\;\text{cha}{\text{r}}_{i}\right)=\sigma \left(s^{r}\left(z_{j},z_{i},r_{k}\right)\right), \end{array}$$

where *σ*(·) represents the sigmoid function. The relation extraction training process is to minimize the cross-entropy of the loss function *L*_re_:

(3) $$\begin{array}{c} L_{\text{re}}=\overset{n}{\underset{i=0}{\sum }}\overset{m}{\underset{j=0}{\sum }}-\text{logPr}\left(\text{head}=y_{i,j},\text{label}=r_{i,j}\;|\;\text{cha}{\text{r}}_{i}\right), \end{array}$$

where *y*_*i*_ belongs to the input token sequence, *r*_*i*_ belongs to the relation set *R*, *y*_*i*_ and *r*_*i*_ stand for the gold annotation of the head and the relation label, and *m* is the number of heads for char_*i*_. Based on equation (2), a threshold would be set after training to decide whether the combination of heads ${\hat{y}}_{i}$ and relation labels ${\hat{r}}_{i}$ should be kept. For the joint learning of entity and relation extraction, the final objective loss would be *L*_ner_ + *L*_re_.

## 5. Results and Discussion

### 5.1. Experiment

All of our models were trained on the environment with two NVIDIA 1080ti GPUs and 12 G RAM. The maximum sequence length was 480. The baseline model BERT-base-Chinese has 12 transformer layers, 768 hidden units, 12 self-attention heads, toltal 110-million parameters. The fine-tuned BERT has the same set and parameter volumes. For the relation extraction model, we used the backpropagation algorithm and the Adam optimizer with an initial learning rate of 0.001 for all RNN layers and 0.00005 for BERT. We apply dropout to the output of each RNN layer; for the baseline model, the dropout probability is 0.5.

### 5.2. Results

As shown in Tables 3 and 4, regardless of the strategies, the joint learning model's recall was superior to that of the pipeline model. These differences were wider as more features were involved. BERT is conceptually simple and empirically powerful, producing superior results on tasks both in pipeline approaches and joint learning models, and especially for the joint learning model, in which F1-measure increased from 0.71 to 0.82. Furthermore, with BERT's participation, the difference between the pipeline approach and the joint learning model enlarged from just below 0.04 to 0.08. The comparison between extracting strategies included pushing the F1-measures to 0.8216 (a 1.1% absolute improvement over the official BERT-base (Chinese), a 10.3% absolute improvement over the baseline models in our experiment with label embedding.

## 6. Discussion

By contrasting various relation extraction methods, we concluded that the joint extraction strategy with pretrained language models and label embedding surpassed the pipeline approaches. The reasons can be summarized as follows:

Firstly, BERT achieves competitive results with joint entity and relation extraction models on all indicators due to its deep bidirectional architecture. This allows the same pretrained model to tackle a specific task with parameters that fine-tune throughout all of the labelled data from the downstream tasks. Simultaneously, each downstream task has separate fine-tuned models, even though they are initialized with the same pretrained parameters. Meanwhile, the RNN-based approaches did not have powerful fitting ability like BERT.

Secondly, compared with pipeline approaches, joint entity recognition and relation extraction improved the F1-measures. This indicated a joint neural model to simultaneously extract entities and relations to avoid the semantic information loss in the CRF layer for the entity recognition task before the sigmoid layer for the relation extraction task. Moreover, the multihead mechanism and label embedding strategy benefits the joint learning model. The multihead selection mechanism enabled an individual entity to participate in multiple relations. Combined with the fact that 16% of the entities in this dataset were involved in more than one relation, we can deduce that the recall of the recognition of overlapping relations has been enhanced. Meanwhile, Table 4 shows that the models without label embedding layers were 0.03~0.06 less on the recall. This means that the embedding of predicted entity labels indeed provided meaningful information for the relation extraction component.

Finally, the fine-tuned pretraining BERT contributed 0.02 more F1-measure to the joint learning model we built. Inspired by other studies [22, 23], we collected nearly 50,000 records, with over 20 million characters of TCM clinical text for the BERT fine-tuning. However, the indicator elevation was limited. We attributed this limitation to the scale of the corpus. As known, clinical data are kept under vigorous supervision to maintain patient privacy. Though affected by this policy, as for future work, we expect to collect TCM medical records in various formats, such as historical literature from ancient dynasties and books published by renowned TCM clinicians.

## 7. Conclusions

As a basic form of patient experienced data, Traditional Chinese Medicine clinical notes record a plethora of symptom-related details. These are instrumental to efficacy evaluation. Due to the custom of presentation, mixture symptom mentions are common in TCM clinical notes, and the conventional symptom extraction method may cause integrity compromise in symptom expression. To cope with this problem, we designed a disassembling and reassembling framework to extract and complete the symptom mentions. The disassembling component is demonstrated in this paper.

We constructed a joint learning model to extract entities and relations from free-text TCM clinical notes simultaneously. Our model comprises a bi-LSTM-CRF layer for the entity recognition task and a sigmoid layer for the relation extraction task. To improve performance, we pretrained the clinical contextualized representation by fine-tuning the BERT on the TCM corpus. Confirmed by the experiment, the proposed multihead extraction model outperformed the baseline method in overlapping relation recognition and automatically captured features without any assistance from exterior NLP tools. This was efficient and convenient for the mixture symptom extraction.

## Figures and Tables

**(a):** 

Type	Counts	Percentage
症状 (symptom)	41,004	56.26%
部位 (area of the body)	26,829	36.8%
程度 (severity)	5,061	6.94%
Total	7,2894	100%

**(b):** 

Type	Counts	Percentage
位于 (located_at)	35,804	86.81%
描述 (is_a_description_of)	5,442	13.19%
Total	41,246	100%

**Table 2:** The frequency at which the entity is involved in relations.

Frequency at which the entity is involved in relations	Counts	Percentage
0	4,706	6.456%
1	56,046	76.886%
2	10,387	14.249%
3	1,440	1.975%
4	241	0.331%
5	65	0.089%
6	7	0.010%
7	3	0.004%

**Table 3:** Joint learning between official BERT-base (Chinese) and fine-tuned BERT.

Model	Label embedding	F1-score	Precision	Recall
BERT-base (Chinese)	Without	0.7968	0.7998	0.7939
	With	0.8102	0.8119	0.8085
Fine-tuned BERT (ours)	Without	0.8016	0.8218	0.7823
	With	0.8216	0.8250	0.8183

**Table 4:** Entity recognition and relation extraction with pipeline approaches.

Model	Label embedding	F1-score	Precision	Recall
Relation extraction pipeline	Without	0.6794	0.8374	0.5716
Multihead joint learning	Without	0.7079	0.8228	0.6212
BERT+relation extraction pipeline	Without	0.7222	0.7596	0.6884
BERT+relation extraction pipeline	With	0.7851	0.8496	0.7297
BERT+multihead joint learning	Without	0.8016	0.8218	0.7823
BERT+multihead joint learning	With	0.8216	0.8250	0.8183

## Data Availability

The clinical notes used in this study were supplied by Prof. Zhang Lei and under license; thus, they cannot be made freely available.
